# Macrophage migration inhibitory factor - a therapeutic target in gallbladder cancer

**DOI:** 10.1186/s12885-015-1855-z

**Published:** 2015-11-04

**Authors:** Tejaswini Subbannayya, Pamela Leal-Rojas, Mustafa A. Barbhuiya, Remya Raja, Santosh Renuse, Gajanan Sathe, Sneha M. Pinto, Nazia Syed, Vishalakshi Nanjappa, Arun H. Patil, Patricia Garcia, Nandini A. Sahasrabuddhe, Bipin Nair, Rafael Guerrero-Preston, Sanjay Navani, Pramod K. Tiwari, Vani Santosh, David Sidransky, T. S. Keshava Prasad, Harsha Gowda, Juan Carlos Roa, Akhilesh Pandey, Aditi Chatterjee

**Affiliations:** 1 Institute of Bioinformatics, International Technology Park, Bangalore, 560066 India; 2 Amrita School of Biotechnology, Amrita University, Kollam, 690525 India; 3 Department of Pathology, Center of Genetic and Immunological Studies (CEGIN) and Scientific and Technological Bioresource Nucleus (BIOREN), Universidad de La Frontera, Temuco, Chile; 4 McKusick-Nathans Institute of Genetic Medicine, Johns Hopkins University School of Medicine, Baltimore, MD 21205 USA; 5 Adrienne Helis Malvin Research Foundation, New Orleans, LA 70130 USA; 6 Manipal University, Madhav Nagar, Manipal, 576104 India; 7 YU-IOB Center for Systems Biology and Molecular Medicine, Yenepoya University, Mangalore, 575018 India; 8 Department of Biochemistry and Molecular Biology, School of Life Sciences, Pondicherry University, Puducherry, 605014 India; 9 School of Biotechnology, KIIT University, Bhubaneswar, Odisha 751024 India; 10 Department of Pathology, Advanced Center for Chronic Diseases (ACCDiS), CITO, Pontificia Universidad Católica de Chile, Santiago, Chile; 11 Department of Otolaryngology-Head and Neck Surgery, Johns Hopkins University School of Medicine, Baltimore, MD 21231 USA; 12 Lab Surgpath, Mumbai, 400034 India; 13 Centre for Genomics, Molecular and Human Genetics, Jiwaji University, Gwalior, 474011 India; 14 School of Studies in Zoology, Jiwaji University, Gwalior, India; 15 Department of Pathology, National Institute of Mental Health and Neurosciences, Bangalore, 560029 India; 16 NIMHANS-IOB Proteomics and Bioinformatics Laboratory, Neurobiology Research Centre, National Institute of Mental Health and Neurosciences, Bangalore, 560029 India; 17 Departments of Biological Chemistry, Johns Hopkins University School of Medicine, Baltimore, MD 21205 USA; 18 Departments of Oncology, Johns Hopkins University School of Medicine, Baltimore, MD 21205 USA; 19 Departments of Pathology, Johns Hopkins University School of Medicine, Baltimore, MD 21205 USA

**Keywords:** Gastrointestinal cancer, RNA interference, Functional inhibition, Suicide substrate, MIF

## Abstract

**Background:**

Poor prognosis in gallbladder cancer is due to late presentation of the disease, lack of reliable biomarkers for early diagnosis and limited targeted therapies. Early diagnostic markers and novel therapeutic targets can significantly improve clinical management of gallbladder cancer.

**Methods:**

Proteomic analysis of four gallbladder cancer cell lines based on the invasive property (non-invasive to highly invasive) was carried out using the isobaric tags for relative and absolute quantitation labeling-based quantitative proteomic approach. The expression of macrophage migration inhibitory factor was analysed in gallbladder adenocarcinoma tissues using immunohistochemistry. *In vitro* cellular assays were carried out in a panel of gallbladder cancer cell lines using MIF inhibitors, ISO-1 and 4-IPP or its specific siRNA.

**Results:**

The quantitative proteomic experiment led to the identification of 3,653 proteins, among which 654 were found to be overexpressed and 387 were downregulated in the invasive cell lines (OCUG-1, NOZ and GB-d1) compared to the non-invasive cell line, TGBC24TKB. Among these, macrophage migration inhibitory factor (MIF) was observed to be highly overexpressed in two of the invasive cell lines. MIF is a pleiotropic proinflammatory cytokine that plays a causative role in multiple diseases, including cancer. MIF has been reported to play a central role in tumor cell proliferation and invasion in several cancers. Immunohistochemical labeling of tumor tissue microarrays for MIF expression revealed that it was overexpressed in 21 of 29 gallbladder adenocarcinoma cases. Silencing/inhibition of MIF using siRNA and/or MIF antagonists resulted in a significant decrease in cell viability, colony forming ability and invasive property of the gallbladder cancer cells.

**Conclusions:**

Our findings support the role of MIF in tumor aggressiveness and suggest its potential application as a therapeutic target for gallbladder cancer.

**Electronic supplementary material:**

The online version of this article (doi:10.1186/s12885-015-1855-z) contains supplementary material, which is available to authorized users.

## Background

Gallbladder cancer (GBC) is a prevalent malignancy of the biliary tract and is the fifth common cancer of the gastrointestinal tract worldwide [1]. In majority of the cases, it manifests at an advanced and unresectable stage [1, 2]. Early detection is incidental, with complete surgical resection of the gallbladder being the only available curative option. The prognosis is dismal with a five-year survival rate of 32 % for lesions confined to the gallbladder mucosa and a one year survival rate of 10 % for advanced stages [2]. To date, various markers including carbohydrate antigen 19–9 (CA19-9) and carcinoembryonic antigen (CEA) have been explored in the diagnosis of GBC. However, these markers lack specificity and sensitivity [3]. Targeted therapy for GBC is limited with bevacizumab which is a vascular endothelial growth factor (VEGF) inhibitor [4]. Apart from bevacizumab, potential therapeutic targets such as estrogen receptor [5], hedgehog signaling [6] and mTOR inhibitors [7] are pending clinical validation. This highlights an immediate need for identification of novel therapeutic targets to improve treatment options and disease outcome.

Mass spectrometry-based proteomic analysis in tandem with isobaric tags for relative and absolute quantitation (iTRAQ) labeling has been employed for the identification of potential biomarkers in several cancers. We have used similar approaches in the past to identify potential biomarkers in esophageal squamous cell carcinoma [8], hepatocellular carcinoma [9] and head and neck squamous cell carcinoma [10]. Similar proteomic strategies have been employed by other groups to identify potential biomarkers in GBC using bile, serum and cell line-based models [11–14]. However, limited effort has been made to identify potential therapeutic targets in GBC. In this study, we used high-resolution mass spectrometry coupled with iTRAQ-based labeling approach to identify proteins which can serve as potential diagnostic markers and/or therapeutic targets. Using a panel of GBC cell lines, we identified a total of 3,653 proteins of which 654 were found to be overexpressed and 387 were downregulated in invasive GBC cell lines as compared to the non-invasive GBC cell line. Amongst these, macrophage migration inhibitory factor (MIF) was found to be overexpressed in two of the invasive GBC cell lines.

MIF is a pro-inflammatory cytokine which plays a key role in innate and adaptive immunity and is associated with inflammatory conditions including cancer. It is secreted by a variety of cells including immune and epithelial cells [15]. MIF has been reported to be overexpressed in multiple cancers, including gastric adenocarcinoma [16], head and neck squamous cell carcinoma [17], esophageal squamous cell carcinoma [18], colorectal [19], pancreatic [20], ovarian [21], and prostate [22] cancers. Knockdown of *MIF* in a murine ovarian cancer cell line, ID8 has been shown to decrease tumor growth and increase the survival in tumor transplanted mice [21]. Similar results were demonstrated in mice grafted with colorectal carcinoma transplants, administered with anti-MIF therapeutics, using either MIF-antibodies or the MIF antagonist (S, R)-3-(4-hydroxyphenyl)-4,5-dihydro-5-isoxazole acetic acid methyl ester (ISO-1) [19]. Pharmacological inhibition of MIF using the MIF irreversible inhibitor, 4-iodo-6-phenylpyrimidine (4-IPP) has shown a decrease in tumor aggressiveness in head and neck squamous cell carcinomas [17] and lung adenocarcinomas [23]. The role of MIF in tumorigenesis has been characterized in other cancers however its function in GBC is yet to be established. In this study, we have assessed the role of MIF as a potential therapeutic target in GBC.

## Methods

### Cell culture

The GBC cell lines, OCUG-1 and NOZ were obtained from Health Science Research Resources Bank, Osaka, Japan. TGBC2TKB, TGBC24TKB and G-415 were purchased from RIKEN Bio Resource Center, Ibaraki, Japan. SNU-308 was obtained from Korean Cell Line Bank, Seoul, Korea. GB-d1 was authenticated by short tandem repeat analysis. The properties and culture conditions of the GBC cell lines, TGBC2TKB, SNU-308, G-415, TGBC24TKB, NOZ, OCUG-1 and GB-d1 are provided in Additional file 1. All cell lines were maintained in humidified incubator with 5 % CO_2_ at 37 °C.

### Protein extraction and iTRAQ labeling

Each cell line was grown to ~80 % confluence, serum starved for 8 h and lysed in 0.5 % SDS-containing buffer. Protein concentration was measured using the BCA method [24]. Equal amount of protein from each cell line was then split into two and treated as technical replicates. Peptides from each sample were differentially labeled using iTRAQ 8-plex reagent (iTRAQ Reagents Multiplex kit, Applied Biosystems/MDS Sciex, Foster City, CA) as described earlier [25]. Briefly, 100 μg of proteins, in replicate, was treated with 2 μl of reducing agent (TCEP, tris (2-carboxyethyl) phosphine) at 60 °C for 1 h and alkylated with 1 μl of cysteine blocking reagent, MMTS (methyl methanethiosulfate) for 10 min at room temperature. Protein samples were digested using sequencing grade trypsin (Promega, San Luis Obispo, CA) at a 1:20 enzyme to protein ratio for 12 h at 37 °C. Peptides from each cell line were labeled with 8 iTRAQ reagents in 60 μl of isopropanol at room temperature as follows – TGBC24TKB (reporter ion m/z 113 and 114), OCUG-1 (reporter ion m/z 115 and 116), NOZ (reporter ion m/z 117 and 118) and GB-d1 (reporter ion m/z 119 and 121). After 2 h, the reaction was quenched by adding 100 μl of water to each sample. The samples were then pooled and vacuum dried.

### Strong cation exchange chromatography

The iTRAQ labeled peptides were fractionated using strong cation exchange chromatography as previously described [8]. Briefly, the pooled iTRAQ-labeled sample was reconstituted with solvent A (10 mM KH_2_PO_4_, 25 % acetonitrile, pH 2.8). The pH of the sample was adjusted to 2.8 using ortho-phosphoric acid. The peptides were loaded onto a PolySULFOETHYL A column (PolyLC, Columbia, MD) (5 μm, 200 Å, 200x 2.1 mm) using Agilent 1260 Infinity series binary HPLC system (Agilent Technologies, Santa Clara, CA). Peptides were loaded at a flow rate of 250 μl/min and washed for 8 min with solvent A. A 35 min gradient from 0 % to 60 % solvent B (350 mM KCl in solvent A, pH 2.8) was used for fractionation. The peptides were detected at a wavelength of 214 nm using a variant wavelength detector module of HPLC system. A total of 96 fractions were collected and further pooled into 24 fractions based on chromatographic peaks. The pooled fractions were vacuum dried and desalted using C_18_ StageTips and stored at −20 °C till further analysis.

### LC-MS/MS analysis

Peptide fractions were analyzed on an LTQ-Orbitrap Velos mass spectrometer (Thermo Scientific, Bremen, Germany) interfaced with Proxeon Easy nLC II system (Thermo Scientific, Bremen, Germany). Peptides were loaded onto trap column (75 μm x 2 cm, Magic C18AQ, 5 μm, 100 Å, Michrom Biosciences Inc., Auburn, CA) using solvent A (0.1 % formic acid) at a flow rate of 3 μl/min and resolved on an analytical column (75 μm x 10 cm, Magic C18AQ, 3 μm, 100 Å, Michrom Biosciences Inc, Auburn, CA) at a flow rate of 350 nl/min using a linear gradient of 7 – 30 % acetonitrile over 80 min. The MS and MS/MS scans were acquired at a mass resolution of 60,000 and 15,000 at 400 m/z, respectively. Full MS scans were acquired in m/z range of 350 – 1800. For each cycle, twenty most abundant precursor ions with charge state ≥2 were sequentially isolated. The fragmentation was carried out using higher energy collision dissociation as the activation method with 40 % normalized collision energy. Isolation width was set to 2 m/z. Singly charged precursor ions and precursors with unassigned charge states were rejected. The acquired ions were dynamically excluded for 45 s. The automatic gain control for full MS and MS/MS was set to 1x10^6^ and 5x10^4^ ions, respectively. The maximum ion accumulation time was set to 100 ms for MS and 300 ms for MS/MS scans. The lock mass option was enabled using polysiloxane ion (m/z, 445.120025) from ambient air for internal calibration as described [26].

### Data analysis

The raw data obtained was processed using Proteome Discoverer (version 1.4) software suite (Thermo Fisher Scientific, Bremen, Germany) and searched using Sequest and Mascot (version 2.2.0, Matrix Science, London, UK) search algorithms against human protein database NCBI RefSeq (Release 63 containing 71,434 protein sequences and known contaminants). The search parameters included: trypsin as the proteolytic enzyme with two missed cleavages allowed, oxidation at methionine as the dynamic modification, alkylation (methylthio) at cysteine and iTRAQ 8-plex modification at N-terminus of the peptide and lysine as static modifications. Precursor and fragment mass tolerance were set to 20 ppm and 0.05 Da, respectively. The peptide and protein data were extracted using high peptide confidence and top one peptide rank filters. The data were also searched against a decoy database to calculate the false discovery rate (FDR). Peptide spectrum matches (PSMs) at 1 % FDR were used for protein identifications. iTRAQ quantitation was done by taking the average of the reporter ion intensities from the technical replicates. The ratios, invasive neoplastic/non-invasive neoplastic, were obtained as follows – 115 + 116 (OCUG-1)/113 + 114 (TGBC24TKB), 117 + 118 (NOZ)/113 + 114 (TGBC24TKB) and 191 + 121 (GB-d1)/113 + 114 (TGBC24TKB).

### Bioinformatics analysis

Proteins identified in this study were classified based on their subcellular localization, molecular function and biological process using Human Protein Reference Database (HPRD; http://www.hprd.org) which is a Gene Ontology (GO) compliant database [27, 28]. The top canonical pathways associated with the differentially expressed proteins in this study were identified through the use of QIAGEN’s Ingenuity Pathway Analysis (IPA®, http://www.qiagen.com/ingenuity).

### Accessibility of proteomic data

The data obtained in this study has been submitted to public repositories to make it accessible to the scientific community. The data on immunohistochemical analysis and the list of proteins and peptides identified has been submitted to Human Proteinpedia [28, 29] (HUPA, http://www.humanproteinpedia.org). The immunohistochemistry (IHC) can be visualised at http://www.humanproteinpedia.org/Experimental_details?exp_id=TE-547399 for cholecystitis and http://www.humanproteinpedia.org/Experimental_details?can_id=105423 for gallbladder adenocarcinoma. The list of proteins and peptides can be accessed at http://www.humanproteinpedia.org/data_display?exp_id=00803. The raw data has been submitted to ProteomeXchange Consortium via the PRIDE public data repository [30] and can be accessed using the data identifier – PXD001566.

### Immunohistochemistry

Tissue microarrays (TMAs) were constructed at Lab Surgpath, Mumbai using the paraffin blocks of gallbladder adenocarcinoma and cholecystitis cases obtained from Cancer Hospital and Research Institute, Gwalior, India with the approval from Institutional Human Ethics Committee and informed consent of the patients. The tissue microarrays were constructed with 29 cases of gallbladder adenocarcinoma and 16 cholecystitis cases. For this, two cores of 2 mm size was taken from each paraffin block and embedded to a recipient paraffin block.

IHC was carried out on both cholecystitis and gallbladder adenocarcinoma cases. A semi-quantitative assessment was performed to evaluate the immunoreactivity as described previously [31]. Briefly, the formalin fixed paraffin embedded tissue sections were deparaffinised and antigen retrieval was carried out using heat-induced epitope retrieval by incubating the slides for 20 minutes in antigen retrieval buffer (0.01 M Trisodium citrate buffer, pH 6). Endogenous peroxidases were quenched using a blocking solution followed by washes with wash buffer (PBS with 0.05 % Tween-20). The sections were incubated with anti-MIF antibody (sc-20121, Santa Cruz Biotechnology, Dallas, TX) at 1:50 dilution overnight at 4 °C in a humidified chamber. The slides were incubated with appropriate horseradish peroxidase conjugated rabbit secondary antibody for 30 minutes at room temperature. Excess secondary antibody was removed using wash buffer followed by addition of DAB substrate. The signal was developed using DAB chromogen (DAKO, Glostrup, Denmark). Tissue sections were then observed under the microscope. The immunohistochemical labeling was assessed by an experienced pathologist. The intensity of staining was scored on a grading scale ranging from 0 to 3+, where 0 represented negative staining, 1+ represented weak staining, 2+ represented moderate staining and 3+ represented strong staining. To determine the statistical significance of MIF expression in gallbladder adenocarcinoma and cholecystitis, Chi-square test was carried out using R version 3.1.0.

### Western blotting

Whole cell extracts of GBC cells, were prepared using modified RIPA lysis Buffer (Merck Millipore, Billerica, MA) containing protease inhibitors (Roche, Indianapolis, IN) and phosphatase inhibitors (Thermo Scientific, Bremen, Germany). Rabbit polyclonal anti-MIF was obtained from Santa Cruz (sc-20121, Santa Cruz Biotechnology, Dallas, TX). β-Actin was used as a loading control. Western blot analysis was performed as previously described [32] using 30 μg protein lysates.

### Processing of conditioned media

Each cell line was grown to ~80 % confluence, washed multiple times with PBS to remove any adherent serum from the cells and then grown in serum-free medium for 8 h. Post-starvation, the conditioned media was collected for each cell line, centrifuged at 800 × g for 10 min to remove any cellular debris. The supernatant was filtered using a 0.22 μm filter (Merck Millipore, Billerica, MA). The filtered supernatant was subsequently concentrated using 3 kDa cut-off filters (Merck Millipore, Billerica, MA). Protein concentration was estimated by BCA assay [24]. Western blot analysis was performed as previously described [32] using 30 μg protein lysates.

### Cell viability assays

The GBC cells were seeded in a 96-well plate at a density of 1x10^4^ cells/well. The cells were vehicle - treated or treated with MIF-antagonist [(S,R)-3-(4-hydroxyphenyl)-4,5-dihydro-5-isoxazole acetic acid methyl ester (ISO-1) (EMD Millipore, Billerica, MA) (0 to 500 μM) or 4-iodo-6-phenylpyrimidine (4-IPP) (Tocris Bioscience, Bristol, UK) (0 to 500 μM) for 48 h in complete medium at 37 °C in 5 % CO_2_ incubator. After 48 h, the medium was aspirated, the cells were rinsed and MTT assays were performed as previously described [33]. All experiments were performed in triplicate.

### siRNA transfection

ON-TARGETplus SMARTpool control siRNA and *MIF* siRNA were purchased from Dharmacon (Lafayette, CO). The GBC cells were transfected with 10 nM of *MIF* siRNA or control siRNA using RNAiMAX (Invitrogen, Carlsbad, CA) according to the manufacturer’s instructions. Transfection was carried out as previously described [32]. Cells were subjected to invasion assay and viability assay 48 h post-transfection, unless otherwise stated.

### Colony formation assays

GBC cell lines were transfected with either *MIF* siRNA or control siRNA. 3x10^3^ cells/well were seeded in 6-well plates. Cell colonies were allowed to grow for 14 days, before the colonies were fixed with methanol and stained with 4 % methylene blue (Sigma, St. Louis, MO). The number of colonies per dish was counted. Similarly, the colony forming ability of the GBC cells were monitored in the presence of MIF antagonists, ISO-1 and 4-iodo-6-phenylpyrimidine (4-IPP). All experiments were performed in triplicate.

### Cell invasion assays

Cell invasion assays were performed in a transwell system using cell culture inserts for 24-well plates with translucent polyethylene terephthalate membrane containing 8 μm pores (BD Biosciences, NJ). The upper compartment of the culture insert was coated with Matrigel (BD Biosciences, San Jose, CA). GBC cells (2x10^4^) were seeded into the transwell chambers in presence of serum-free medium. Complete media was added to the lower compartment and the cells were incubated at 37 °C in 5 % CO_2_ incubator for 48 h. Post-incubation, the upper surface of the membrane was wiped with a cotton-tip applicator to remove non-migratory cells. Cells that migrated to the lower side of membrane were fixed and stained using 4 % methylene blue (Sigma, St. Louis, MO). The number of invaded cells was counted using a light microscope. All experiments were done in duplicates and repeated thrice.

### Statistical analysis

Paired t-test was carried out to evaluate the difference between control and treated groups. *P* ≤ 0.05 was considered to indicate statistical significance.

## Results

### Quantitative mass spectrometric analysis of GBC cell proteome

**Fig. 1:**
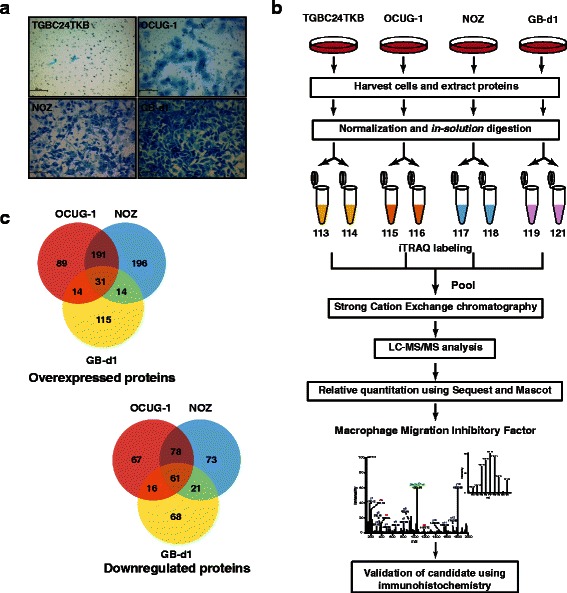
Experimental design and proteomic result**a** Invasive property of GBC cell lines - TGBC24TKB - non-invasive.; OCUG-1 - moderately invasive; NOZ – moderately invasive; GB-d1- highly invasive. **b** Workflow for quantitative proteomic analysis of GBC cell line using iTRAQ labeling. **c** Venn diagrams depicting the overlap of the differentially expressed proteins in the three invasive cell lines, OCUG-1, NOZ and GB-d1

Four GBC cell lines (TGBC24TKB, OCUG-1, NOZ and GB-d1) were selected to study the GBC cell proteome based on their invasive abilities. Of the four cell lines, TGBC24TKB was non-invasive. OCUG-1, NOZ and GB-d1 had varied invasive ability ranging from moderate to highly invasive (Fig. 1a). The experimental workflow used in this study is depicted in Fig. 1b. The resulting MS/MS data was searched against Human RefSeq 63 protein database using Sequest and Mascot search algorithms through Proteome Discoverer platform suite. A total of 3,653 proteins were identified. Of these, 654 proteins were found to be overexpressed (≥2-fold) and 387 were downregulated (≤2-fold). Among these, 31 were found to be overexpressed and 61 were found to be downregulated in all the three invasive GBC cell lines (Fig. 1c). The complete list of proteins and peptides obtained is provided in the Additional files 2 and 3. The list of the differentially expressed proteins is provided in Additional files 4 and 5.

Earlier studies in GBC have reported the dysregulation of CD44 antigen (*CD44*), matrix metallo peptidase 1 (*MMP1*) and cadherin-1 (*CDH1*) [34–36]. However, to our knowledge there are no reports of these proteins in high-throughput mass spectrometry data in GBC. In addition to the above mentioned molecules, this study has also identified proteins which have not been previously described in context of GBC, such as macrophage migration inhibitory factor (*MIF*), caldesmon (*CALD1*), plakophilin (*PKP2*) and desmocollin (*DSC2*) however have been reported earlier in gastrointestinal cancers. A partial list of these proteins is given in Table 1.

**Table 1:** Partial list of differentially expressed proteins identified in GBC

Differentially expressed proteins not previously reported in GBC						
Gene symbol	Protein name	Function		Fold change		
				OCUG-1/ TGBC 24TKB	NOZ/ TGBC 24TKB	GB-d1/ TGBC 24TKB
MIF	Macrophage migration inhibitory factor	Pro-inflammatory cytokine		3.9	4.9	1.4
CALD1	Caldesmon	Calmodulin-binding protein		3.6	1.9	4.2
DSC2	Desmocollin-2	Calcium-dependent glycoprotein required for cell adhesion and desmosome formation		0.2	0.2	0.3
PKP2	Plakophilin-2	Cell adhesion molecule involved in linking cadherins to intermediate filaments in the cytoskeleton		0.4	0.5	0.4
Differentially expressed proteins previously reported in GBC						
Gene symbol	Protein name	Function	Fold change			Citation
			OCUG-1/ TGBC 24TKB	NOZ/ TGBC 24TKB	GB-d1/ TGBC 24TKB	
CD44	CD44 antigen	Cell-cell interactions, cell adhesion and migration; cancer stem cell marker	2.2	3.0	2.2	Ylagan et. al., 2000 [34]
MMP1	Matrix metallo peptidase 1	Breakdown of extracellular matrix	2.7	2.1	2.5	Du et. al.,2011 [35]
CDH1	Cadherin-1	Cell adhesion, epithelial cell marker	0.2	0.3	0.5	Hirata et. al., 2006 [36]

Bioinformatics analysis of all the proteins identified in this study was carried out to categorize them based on the subcellular localization, molecular function and biological processes (Additional file 6a, 6b and 6c). The classifications were based on annotations in the Human Protein Reference Database (HPRD) [27]. This analysis revealed that 27 % of the proteins identified in this study localized to the nucleus and 23 % localized to the cytoplasm. To gain insights into the altered pathways in GBC, network analysis was performed using the differentially expressed proteins (2-fold cut-off) in the invasive cell lines compared to the non-invasive cell line used in this study. The top canonical pathways identified using Ingenuity database are depicted under Additional file 6d, which includes integrin signaling and epithelial adherens junction signaling. Previous studies using mice fibroblasts indicate that cellular adhesion leads to activation of PKC resulting in the secretion of MIF. This, in turn, promotes integrin-mediated activation of MAP kinase and cell cycle progression [37].

**Fig. 2:**
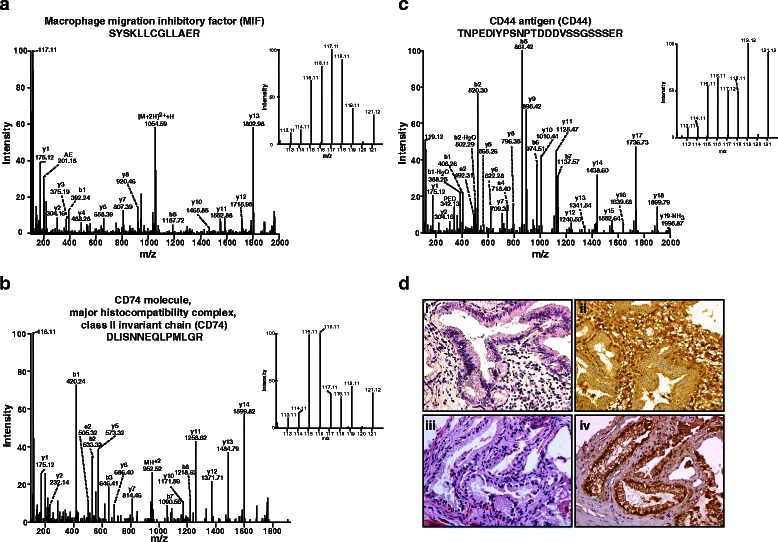
Representative MS/MS spectra of overexpressed proteins and validation of MIF by immunohistochemistry. Representative MS/MS spectra of overexpressed proteins in invasive GBC cell lines, OCUG-1, NOZ and GB-d1 as compared with the non-invasive GBC cell line, TGBC24TKB. **a** Macrophage migration inhibitory factor (MIF). **b** CD74 molecule, major histocompatibility complex, class II invariant chain (CD74). **c** CD44 antigen (CD44). **d** Validation of MIF by IHC. Representative sections from cholecystitis tissues (weak staining) – (i) stained with hematoxylin and eosin; (ii) probed with anti-MIF antibody. Representative sections from gallbladder adenocarcinoma tissue (strong staining) - (iii) stained with hematoxylin and eosin; (iv) probed with anti-MIF antibody

MIF, one of the novel proteins identified by us in this study, was found to be overexpressed >3-fold in two of the invasive GBC cell lines and was considered for further validation. Apart from MIF, the proteins related to the MIF nexus identified in our study are depicted under Additional file 7. This signaling network of MIF and its associated molecules were identified through literature survey. Representative MS/MS spectra of a subset of peptides identified for MIF and associated molecules such as CD74 and CD44 are shown in Figs. 2a, 2b and 2c.

### Immunohistochemical validation of MIF in neoplastic and non-neoplastic gallbladder tissue

Since MIF was found to be overexpressed >3-fold in two of the invasive GBC cell lines, we studied the expression of MIF in primary GBC tissue using immunohistochemical staining. MIF, being a secretory molecule, shows both cytoplasmic and extracellular localization. Tissue microarray-based immunohistochemical validation was carried out using 29 GBC and 16 cholecystitis tissues. A variable staining pattern was noted across cases of gallbladder adenocarcinoma and cholecystitis. About 72 % (21 of 29) of gallbladder adenocarcinoma cases showed moderate to strong staining (2+ to 3+) while 62 % (10 of 16) of the cholecystitis cases showed negative to weak staining (0 to 1+). Notably, none of the cholecystitis cases showed 3+ staining. A Chi-square test clearly indicated a significant overexpression of MIF in gallbladder adenocarcinoma cases (*p*-value <0.05) at a confidence level greater than 95 %. The results of the immunohistochemical validation are provided in Table 2. MIF was observed to be predominantly localized in the cytoplasm. The representative staining patterns for strong MIF staining (3+) in GBC and weak MIF staining (1+) in cholecystitis tissues are illustrated in Fig. 2d. The representative staining patterns for weak MIF staining (1+) in GBC and moderate MIF staining (2+) in cholecystitis tissues are illustrated in Additional file 8.

**Table 2:** Summary of the immunohistochemical validation for MIF in GBC

Staining Intensity	Cholecystitis	Gallbladder adenocarcinoma
0 – 1+ (Negative – Weak)	10	8
2+ − 3+ (Moderate – Strong)	6	21
*p*-value of significance	2.2E-02	
Subcellular location of staining	Predominantly cytoplasmic	

### MIF affects the colony forming ability of the GBC cells

**Fig. 3:**
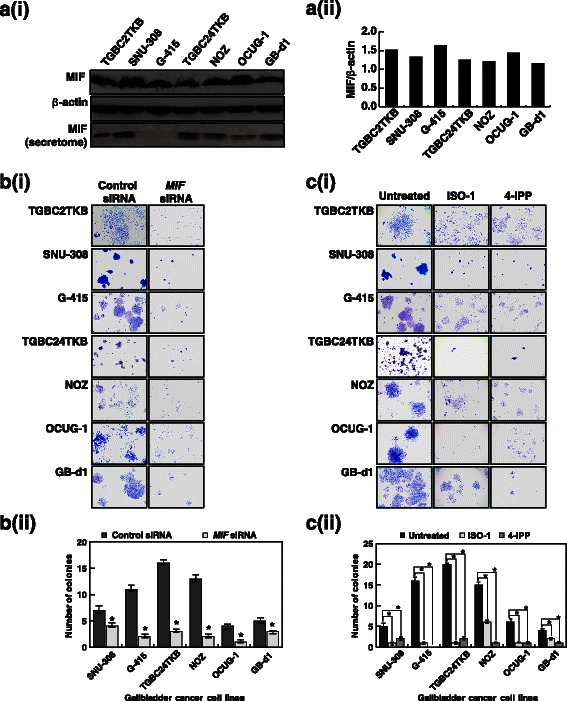
MIF affects the colony forming ability of the GBC cells – **a**-**i** Expression of MIF across a panel of GBC cell lines. Western blot analysis was performed using anti-MIF antibody. β-Actin was used as loading control. Top panel: GBC whole cell lysates, Middle panel: β-Actin, Bottom panel: GBC cell secretome. **a**-**ii** A graphical representation of MIF expression in GBC cell lines compared to β-Actin. **b**-**i** Colony forming ability of GBC cell lines was decreased post-transfection with MIF siRNA. **b**-**ii** A graphical representation of the same *P < 0.05. **c**-**i** Inhibition of MIF in GBC cell lines with ISO-1 (50 μM) and 4-IPP (5 μM) led to a decrease in the colony forming ability of the cells. **c**-**ii** A graphical representation of the same *P < 0.05

Having found that MIF is overexpressed in gallbladder adenocarcinoma tissue, we sought to investigate the role of MIF in GBC. MIF has been reported to be overexpressed in multiple cancers and its role in tumor cell proliferation has been documented [17, 18, 20, 22]. We assessed the expression of MIF in a panel of GBC cell lines (TGBC2TKB, SNU-308, G-415, TGBC24TKB, NOZ, OCUG-1 and GB-d1) and found detectable heterogeneous expression of the protein in all cell lines (Fig. 3a). The Western blot analysis of the MIF expression correlated well with the mass spectrometry results. Since MIF is a secretory protein, we checked the expression of MIF in the secretome of all the GBC cell lines (Fig. 3a). We observed a varied expression of MIF in the secretome of the six GBC cell lines. MIF was below detectable limits in the secretome of G-415. Western blot analysis revealed a significant decrease in the endogenous expression of MIF using MIF siRNA in all the cell lines (Additional file 9a).

We examined the colony formation and invasion ability of the GBC cells used in this study. As shown in Additional file 9b, all GBC cells except TGBC2TKB grow in defined colonies. The colonies of TGBC2TKB cells, that are less clearly defined than those of other GBC cells, indicate cell scattering as is evidenced by the invasion data (Additional file 9b). A comparison of the colony forming and invasive ability of the GBC cells revealed a heterogeneous pattern (Additional file 9c) with no direct association with the MIF expression. As this does not rule out the importance of MIF in the oncogenic potential of the GBC lines, we next studied the role of MIF in the cell survival and invasive ability of the GBC cells.

To determine whether inhibition of MIF had any effect on cell survival, we attempted to silence the expression of MIF in the GBC cell lines using *MIF*-specific siRNA. Colony formation assays are often carried out to analyze the ability of cells to grow infinitely contributing to its oncogenic potential. Our results indicate that siRNA-mediated silencing of *MIF* in a panel of GBC cell lines resulted in a significant decrease in the colony forming ability of the cells (*p*-value <0.05) (Fig. 3b). Using an alternative strategy, GBC cell lines were treated with ISO-1, a MIF antagonist [38] and 4-IPP, a MIF irreversible inhibitor [23]. GBC cell lines showed decreased cell viability in the presence of the MIF irreversible inhibitor, 4-IPP compared to the MIF antagonist ISO-1 (Additional file 10a and 10b). Akin to the effect of siRNA-mediated silencing over the colony forming ability, GBC cell lines showed a significant reduction in their colony forming ability in the presence of either 50 μM ISO-1 or 5 μM of 4-IPP (*p*-value <0.05) (Fig. 3c).

### Inhibition of MIF decreases the invasive property of the GBC cells

**Fig. 4:**
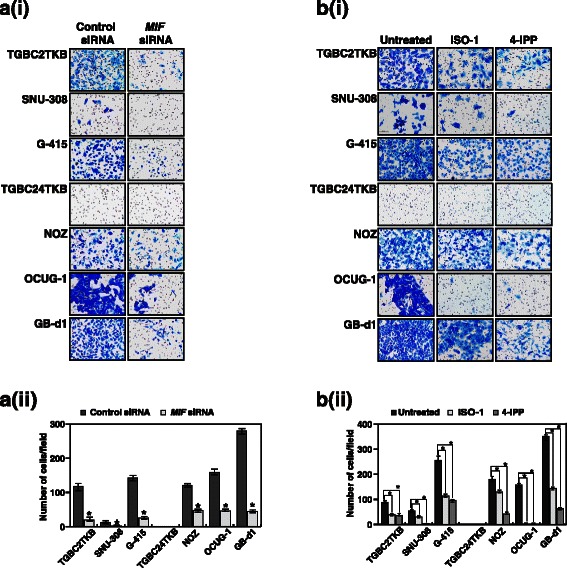
Inhibition of MIF decreases the invasive property of the GBC cells. **a**-**i** siRNA mediated silencing of MIF decreases the invasive property of GBC cells. **a**-**ii** A graphical representation of the same *P < 0.05. **b**-**i** Inhibition of MIF using ISO-1 (50 μM) and 4-IPP (5 μM) lead to a decrease in the invasive ability of the GBC cells. **b**-**ii** A graphical representation of the same *P < 0.05

Having observed that inhibition of MIF leads to a decrease in the colony formation ability of the GBC cell lines, we addressed whether MIF has a potential role in GBC metastasis. GBC cell lines were used as an in vitro model to study their invasive property whereby the endogenous expression of *MIF* was silenced using its specific siRNA and its functional activity was inhibited using MIF inhibitors, ISO-1 and 4-IPP. siRNA-mediated silencing of MIF resulted in a significant decrease in the invasive ability of the cells (*p*-value <0.05) (Fig. 4a). In agreement with the colony formation assays, treatment of GBC cell lines with 5 μM of 4-IPP resulted in a significant decrease in the invasive ability of the cells (*p*-value <0.05) (Fig. 4b). Taken together, these results indicate that 4-IPP is more potent inhibitor of MIF in GBC. These results suggest that inhibition/silencing of MIF can remarkably decrease the ability of the GBC cells to invade the extracellular matrix.

## Discussion

Early diagnosis and treatment of GBC requires elucidation of molecular events associated with tumor progression and aggressiveness in GBC. Mass spectrometry has emerged as a reliable tool to identify differentially regulated proteins across different conditions enabling the discovery of potential biomarkers and therapeutic targets in cancer. In this study, quantitative proteomic analysis of a panel of GBC cell lines led to the identification of more than 1,000 differentially expressed proteins - 654 of which were found to be overexpressed and 387 were downregulated in invasive GBC cell lines. MIF, a pro-inflammatory cytokine, was found to be overexpressed >3-fold in two of the invasive GBC cell lines as compared to the non-invasive cell line, TGBC24TKB.

Tumor growth and metastasis is often accompanied by chronic inflammation, a condition commonly observed in cholecystitis and in the development of GBC. The ability of MIF to suppress anti-inflammatory pathways makes it a molecule of choice to be investigated for such conditions. MIF enhances its activity by inducing other inflammatory cytokines including TNF-alpha and IL-1 [39]. MIF has been reported to act as an antagonist of glucocorticoids regulating its anti-inflammatory effects [15]. MIF exerts its effects via the CD74/CD44 receptor complex. MIF has also been reported to activate the chemokine receptors CXCR2 and CXCR4 [40] to exert its chemokine-like function. Overexpression of MIF has been reported in multiple human cancers [17, 18, 20, 22]. MIF contributes to tumor development, progression and tumor cell survival through inhibition of p53-mediated apoptosis. This is achieved through the sustained activation of the ERK signaling pathway [41]. MIF also exerts its pro-survival and anti-apoptotic effects through the activation of PI3K/Akt cascade [42]. MIF causes an increased transcription of cyclin D leading to hyperphosphorylation of Rb and hence augmenting cellular proliferation [43]. Recent studies indicate that MIF leads to HIF-1α activation under hypoxic conditions leading to enhancement of cancer growth and metastasis [44]. In addition, MIF has been suggested as a potential biomarker for hepatocellular carcinoma, colorectal cancer, gastric cancer and non-melanoma skin cancer [45]. CD74, which acts as a receptor to MIF, was also found to be overexpressed in two of the GBC cell lines used in the proteomic experiment in this study. The expression of CD74 has been linked to several cancers [46]. The co-receptor of MIF, CD44 was also found to be overexpressed in all the GBC cell lines. Long-term activation of CD44 has been reported to play a key role in tumor progression [34].

Studies have shown that vaccination of human subjects with autologous tumor cells modified to secrete granulocyte-macrophage colony stimulating factor (GM-CSF) and antibody-based blockade of cytotoxic T-lymphocyte-associated antigen-4 (CTLA4) results in a humoral response against multiple angiogenic cytokines, including MIF. This antibody-based inhibition of MIF attenuates macrophage Tie-2 (TEK) expression and matrix metalloproteinase-9 (MMP9) production. This and studies by others indicate that blockade of VEGF, angiopoietins, and MIF may be effective in tumor regression [47, 48]. Taken together, these findings suggest that MIF can be explored as a therapeutic target in GBC.

For a molecule to act as a therapeutic target it is essential that it has to be expressed in the cancer tissue. In this study, tissue microarray-based immunohistochemical staining revealed overexpression of MIF in more than 72 % of the gallbladder adenocarcinoma cases. These findings suggest MIF as a potential therapeutic target in GBC.

ISO-1 is an antagonist of MIF which binds to the hydrophobic catalytic pocket of MIF and inhibits its tautomerase activity thereby counteracting glucocorticoid-inhibited TNF release as well as inhibiting the cytokine action of MIF on PLA2 activity. Inhibition of MIF using ISO-1 has been demonstrated to provide protection from septic shock induced by endotoxins [38]. *In vitro* and *in vivo* MIF inhibition using its specific inhibitor has been shown to be potentially effective in multiple cancers [19, 23, 49–54]. In our study, knockdown of endogenous MIF expression using ISO-1 or its specific siRNA showed a significant decrease in cellular proliferation, invasion and colony forming ability of GBC cell lines. As evidenced from the current study and in agreement with literature, relatively high concentrations of ISO-1 are potentially effective in rendering cellular death. This property of ISO-1 has hindered the use of this antagonist in clinical settings. Meanwhile, the small molecule inhibitor 4-IPP has been reported to be ~5-10 times more potent than the MIF antagonist, ISO-1. The antagonist, 4-IPP acts as a suicide substrate to MIF through covalently modifying the catalytically active N-terminal proline [23]. In this study, we demonstrate that the inhibition of MIF activity using 4-IPP decreased cellular proliferation, invasion and colony forming ability of GBC cell lines and was more potent than the prototypic MIF antagonist, ISO-1. Taken together, these studies provide experimental evidence of the potency of the MIF inhibitors in multiple cancers including GBC. We suggest that targeted MIF therapy might be effectively combined with antibody-based therapy to improve patient outcome in other cancers including GBC. Further clinical investigations of these inhibitors are needed to establish their role as a therapeutic target in cancer.

## Conclusions

Our data suggests that MIF is active in GBC and plays a pivotal role in cellular proliferation and invasiveness of GBC. Our current study does not rule out the role of other molecules and/or genomic factors towards tumor progression of GBC. The findings of this study and those of others elucidates the role of MIF as a therapeutic target in multiple cancers including GBC. Further studies are warranted to confirm our findings in clinical settings.

## Additional files

Additional file 1: **Properties of GBC cell lines used in the study.** (XLS 27 kb)
Additional file 2: **Proteins identified in GBC cell lines using Sequest and Mascot search algorithm.** (XLS 1180 kb)
Additional file 3: **Peptides identified in GBC cell lines using Sequest and Mascot search algorithm.** (XLS 5659 kb)
Additional file 4: **Overexpressed proteins identified in invasive GBC cell lines.** (XLS 406 kb)
Additional file 5: **Downregulated proteins identified in invasive GBC cell lines.** (XLS 277 kb)
Additional file 6: **Gene ontology-based classification of proteins identified in the study.** (**a**) Subcellular localization (**b**) Molecular function (**c**) Biological process. (**d**) Graphical representation of the top 12 canonical pathways of the differentially expressed proteins identified in this study generated using Ingenuity Pathway Analysis. The columns represent the –log of the *p*-value for all of the genes in each particular canonical pathway family calculated based on Fisher’s exact test. The line graph represents the ratio plot indicating the number of genes differentially expressed in this study relative to the total number of genes in that particular canonical pathway. (PDF 311 kb)
Additional file 7: **A graphical representation of the proteins related to the MIF nexus identified in our study.** (PDF 764 kb)
Additional file 8: **Representative images of MIF by immunohistochemistry.** Representative sections from cholecystitis tissues (moderate staining) – (i) stained with hematoxylin and eosin; (ii) probed with anti-MIF antibody. Representative sections from gallbladder adenocarcinoma tissue (weak staining); (iii) stained with hematoxylin and eosin; (iv) probed with anti-MIF antibody. (PDF 1714 kb)
Additional file 9: **(a) Knockdown of*****MIF*****using its specific siRNA in a panel of GBC cell lines. Western blot analysis was performed using anti-MIF antibody.** β-Actin was used as a loading control. (**b**) Colony forming ability and invasive property of GBC cell lines. (**c**) Scatter plot representing invasive ability versus the colony forming ability of GBC cell lines. (PDF 1399 kb)
Additional file 10: **Cell viability of GBC cell lines was measured by MTT assay after treatment with indicated concentrations of ISO-1 (a) and 4-IPP (b) for 48 h.** (PDF 769 kb)

## Abbreviations

MIF: Macrophage migration inhibitory factor
iTRAQ: Isobaric tags for relative and absolute quantitation
GBC: Gallbladder cancer
ISO-1: (S, R)-3-(4-hydroxyphenyl)-4,5-dihydro-5-isoxazole acetic acid methyl ester
4-IPP: 4-iodo-6-phenylpyrimidine
IHC: Immunohistochemistry
