# Supplementary figures and images for: Macrophage migration inhibitory factor - a therapeutic target in gallbladder cancer
Source: BMC Cancer. 2015 Nov 4;15:843. doi: 10.1186/s12885-015-1855-z (PMC4632274; doi:10.1186/s12885-015-1855-z)

a

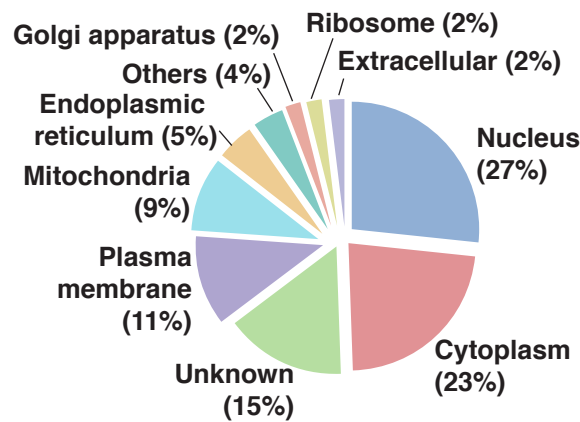

b

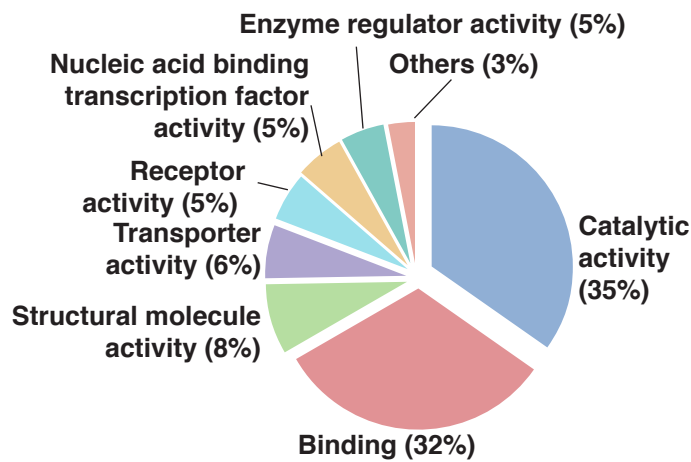

c

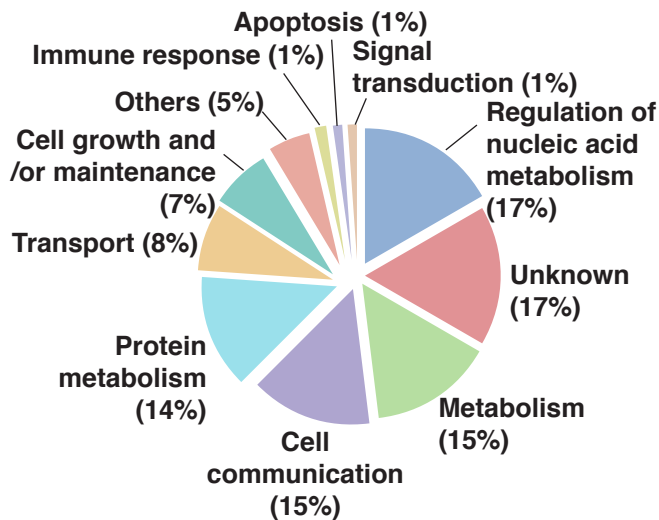

d

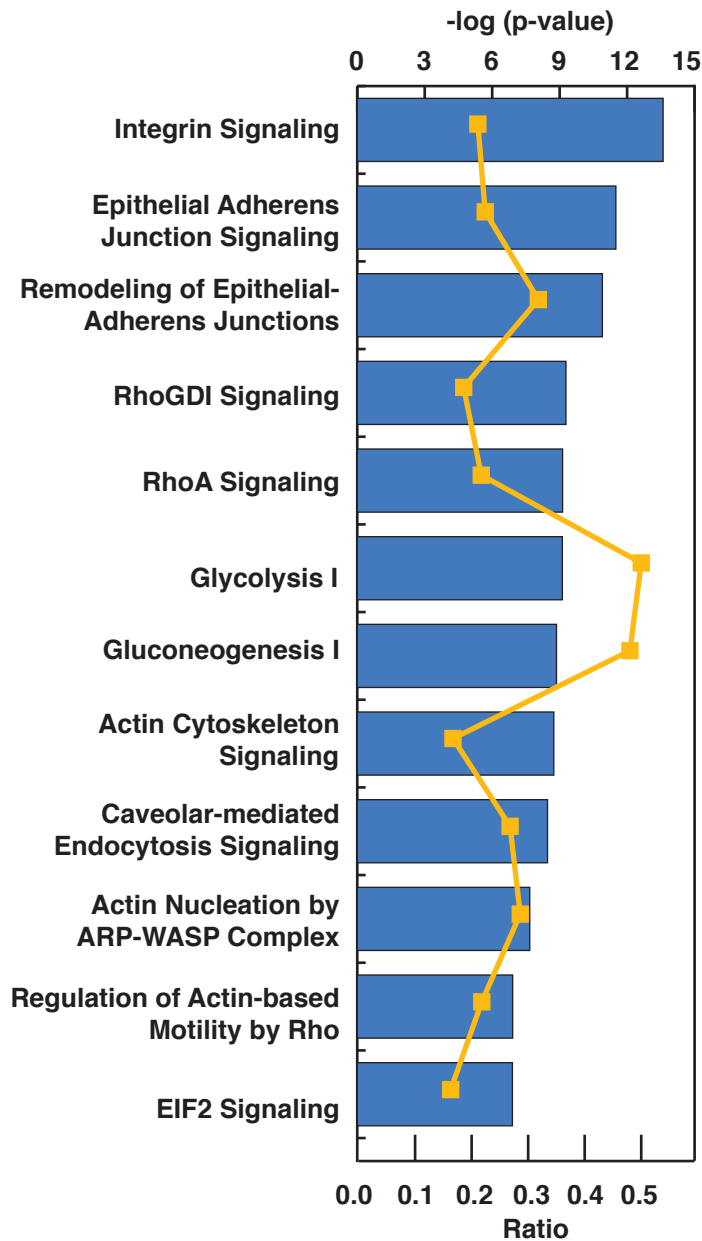

Supplement: Additional file 6: — Gene ontology-based classification of proteins identified in the study. (a) Subcellular localization (b) Molecular function (c) Biological process. (d) Graphical representation of the top 12 canonical pathways of the differentially expressed proteins identified in this study generated using Ingenuity Pathway Analysis. The columns represent the –log of the p-value for all of the genes in each particular canonical pathway family calculated based on Fisher’s exact test. The line graph represents the ratio plot indicating the number of genes differentially expressed in this study relative to the total number of genes in that particular canonical pathway. (PDF 311 kb) [file 12885_2015_1855_MOESM6_ESM.pdf]

# Additional File 8

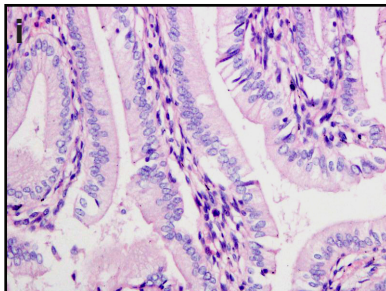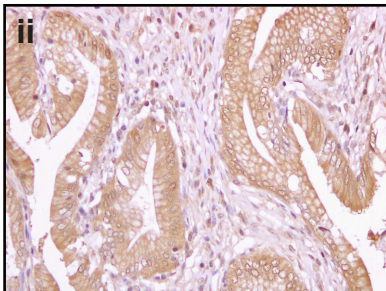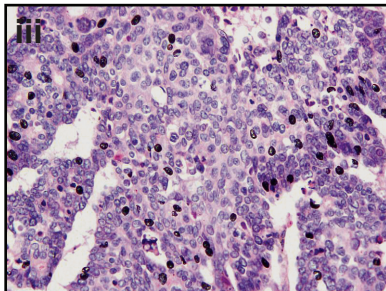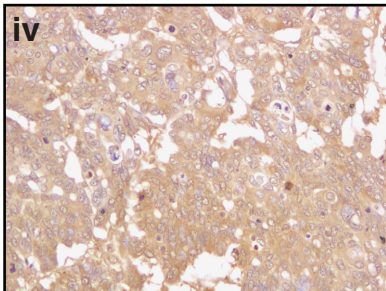

Supplement: Additional file 8: — Representative images of MIF by immunohistochemistry. Representative sections from cholecystitis tissues (moderate staining) – (i) stained with hematoxylin and eosin; (ii) probed with anti-MIF antibody. Representative sections from gallbladder adenocarcinoma tissue (weak staining); (iii) stained with hematoxylin and eosin; (iv) probed with anti-MIF antibody. (PDF 1714 kb) [file 12885_2015_1855_MOESM8_ESM.pdf]

**a**

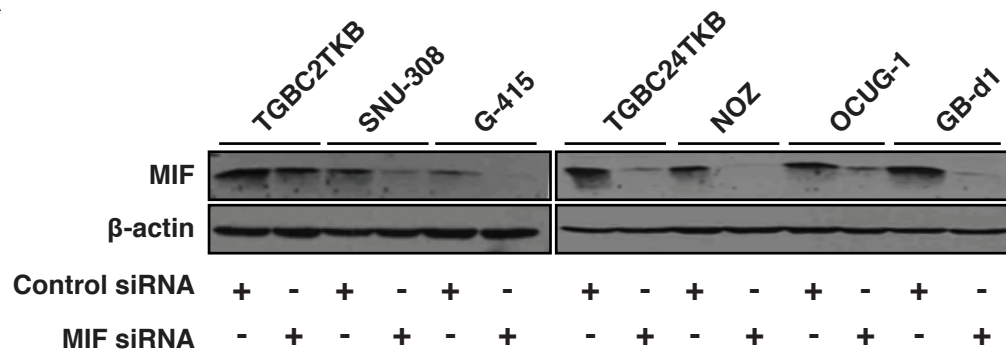

**b**

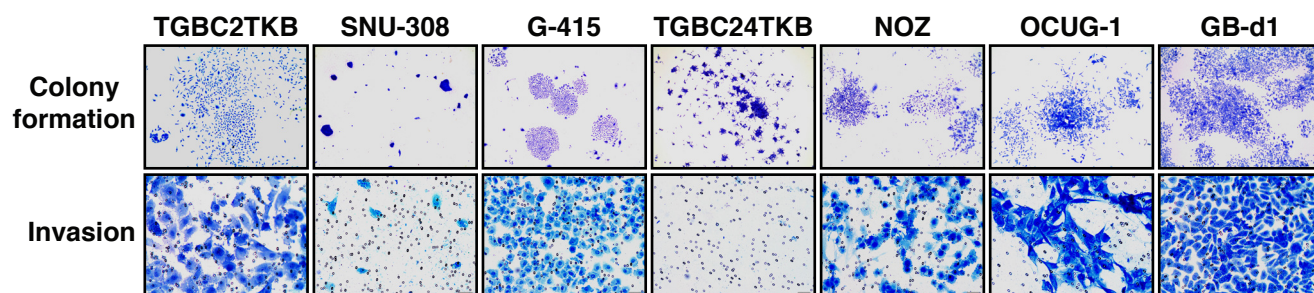

**c**

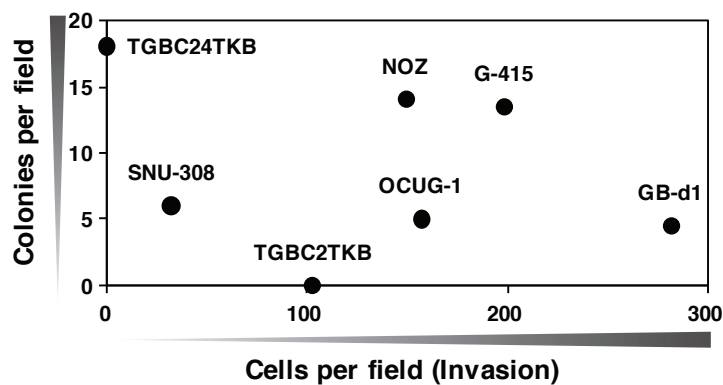

Supplement: Additional file 9: — (a) Knockdown ofMIFusing its specific siRNA in a panel of GBC cell lines. Western blot analysis was performed using anti-MIF antibody. β-Actin was used as a loading control. (b) Colony forming ability and invasive property of GBC cell lines. (c) Scatter plot representing invasive ability versus the colony forming ability of GBC cell lines. (PDF 1399 kb) [file 12885_2015_1855_MOESM9_ESM.pdf]

# Additional File 10

**a**

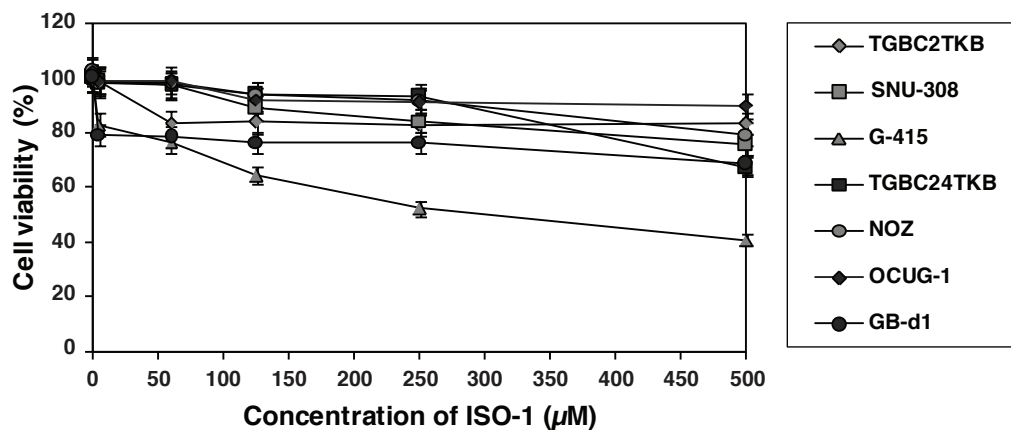

**b**

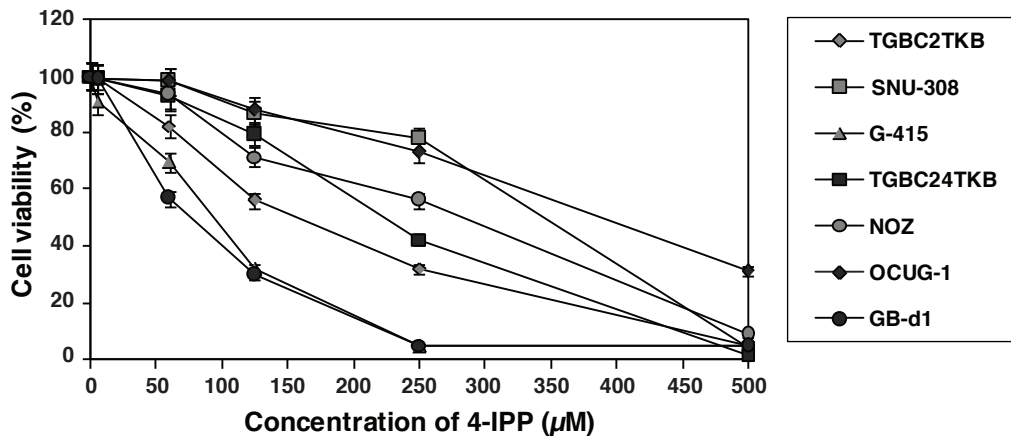

Supplement: Additional file 10: — Cell viability of GBC cell lines was measured by MTT assay after treatment with indicated concentrations of ISO-1 (a) and 4-IPP (b) for 48 h. (PDF 769 kb) [file 12885_2015_1855_MOESM10_ESM.pdf]
