# Supplementary material for: Macrophage migration inhibitory factor - a therapeutic target in gallbladder cancer
Source: BMC Cancer. 2015 Nov 4;15:843. doi: 10.1186/s12885-015-1855-z (PMC4632274; doi:10.1186/s12885-015-1855-z)
Supplement: Additional file 7: — A graphical representation of the proteins related to the MIF nexus identified in our study. (PDF 764 kb) [file 12885_2015_1855_MOESM7_ESM.pdf]

Additional File 7

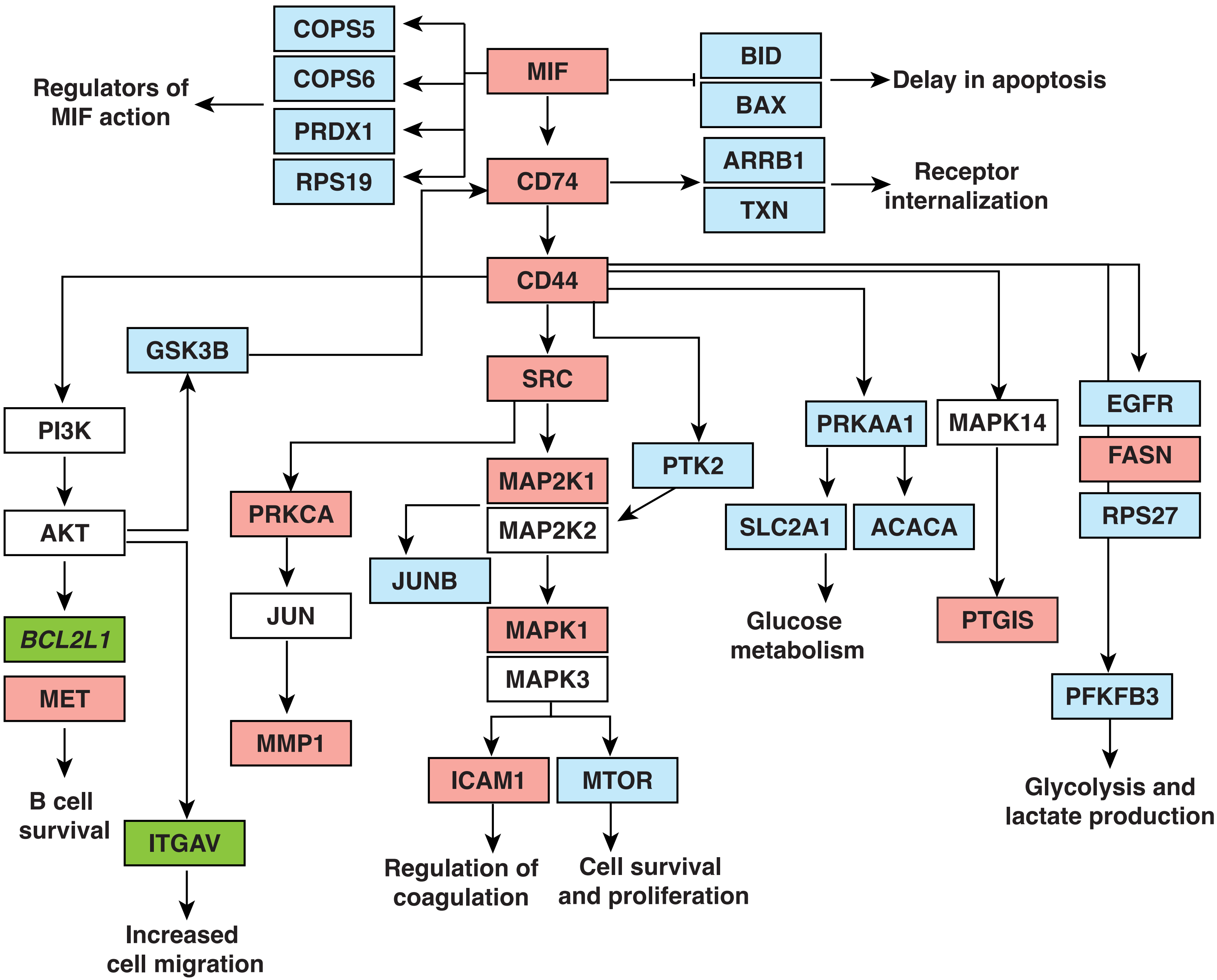

Legend

- Proteins overexpressed in this study
- Proteins downregulated in this study
- Proteins unchanged in this study
- Proteins not identified in this study
